# Acute adverse reactions after multiple initially well-tolerated gadolinium-based contrast-enhanced abdomen MRIs in pediatric patients

**DOI:** 10.1371/journal.pone.0313495

**Published:** 2024-12-03

**Authors:** Azadeh Hojreh, Amra Mulabdic, Andreas Heilos, Andreas Peyrl, Katharina Lampichler, Marcus Raudner, Dietmar Tamandl, Ahmed Ba-Ssalamah, Zsolt Szepfalusi

**Affiliations:** 1 Department of Biomedical Imaging and Image-guided Therapy, Medical University of Vienna, Vienna, Austria; 2 Division of Pediatric Nephrology and Gastroenterology Department of Pediatrics and Adolescent Medicine, Comprehensive Center for Pediatrics, Medical University of Vienna, Vienna, Austria; 3 Division of Neonatology, Pediatric Intensive Care Medicine and Neuropediatrics, Department of Pediatrics and Adolescent Medicine, Comprehensive Center for Pediatrics, Medical University of Vienna, Vienna, Austria; 4 Division of Pediatric Pulmonology, Allergology and Endocrinology, Department of Pediatrics and Adolescent Medicine, Comprehensive Center for Pediatrics, Medical University of Vienna, Vienna, Austria; Affiliated Hospital of Nanjing University of Chinese Medicine: Jiangsu Province Academy of Traditional Chinese Medicine, CHINA

## Abstract

**Purpose:**

Repeated gadolinium-based contrast agent (GBCA)-enhanced MRIs are crucial in the diagnosis and follow-up of oncologic and chronic disorders in pediatric patients. The aim of the study was to evaluate the frequency and severity of adverse reactions to GBCAs in children after a single vs. multiple GBCA-enhanced abdomen MRIs.

**Material and methods:**

All pediatric patients with at least one GBCA-enhanced abdominal MRI between 2009 and 2020 were retrospectively evaluated based on adverse reactions reports, according to the classification system of the American College of Radiology and guidelines on contrast agents of the European Society of Urogenital Radiology. A Student´s t-test analysis, a spearman ρ-correlation and a Chi-square test between the reported adverse reactions and the total number of GBCA applications, and the number of each applied GBCA was calculated. A p-value <0.05 was considered significant.

**Results:**

Of 623 patients with 964 GBCA-enhanced abdomen MRIs, there were 464 patients with only one and 159 patients with multiple GBCA administrations. Of 964 GBCA doses administrated, two cases with urticaria (mild allergy-like adverse reaction) and one case with vomiting (mild chemotoxic adverse reaction) were recorded (3/964 = 0.31%), but all the reports were in patients with multiple GBCA administration (3/159 = 1.89%). No adverse reactions in patients with a single GBCA administration were observed. The reported adverse reactions correlated significantly with the total number of GBCAs (p<0.001) and the number of each GBCA (p<0.001 or p = 0.002). The independent two-tailed t-tests, and the chi-square test were significant (p<0.001, p = 0.003).

**Conclusion:**

GBCA-associated adverse reactions are rare and mostly mild, but initially well-tolerated GBCA could cause adverse reactions due to the increase likelihood of drug hypersensitivity upon repeated GBCA exposure.

## Introduction

Gadolinium-based contrast agent (GBCA)-enhanced MR scans improve the efficacy of diagnostic imaging and they are frequently used to follow-up pediatric patients with oncological [1–5], chronic inflammatory [6, 7], hepato-pancreato-biliary [8, 9], kidney, and urogenital diseases [10, 11], due to the increased lesion-to-parenchyma contrast [12]. They are also used in cardiovascular [13] and in functional imaging [8, 9, 14, 15].

GBCAs have been approved and are well tolerated by most patients and acute adverse reactions to GBCAs are rare [16]. Acute adverse reactions (AR) to GBCA are categorized as either allergic-like or chemotoxic, and they are classified into three severities (mild, moderate, or severe), according to the classification system of the American College of Radiology (ACR) [16] and guidelines on contrast agents of the European Society of Urogenital Radiology (ESUR) [17]. Mild reactions, such as mild urticaria, flushing, nausea, vomiting, and headache are usually of short duration and self-limiting and generally do not need any specific treatment [16, 17]. Moderate adverse reactions show more serious manifestations of the same symptoms, as well as moderate degrees of hypotension and bronchospasm [16, 17]. Severe, life-threatening reactions can include all the symptoms described as minor and moderate, plus convulsions, unconsciousness, laryngeal edema, severe bronchospasm, pulmonary edema, severe cardiac dysrhythmias, and cardiac arrest [16, 17]. The frequency of allergic-like reactions varies between 0.004%–0.7% [16]; severe life-threatening anaphylactic reactions [16, 18–23], and fatal reactions to GBCAs have also been reported [24]. At clinical doses (0.1–0.2 mmol/kg for most GBCA), the incidence of adverse reaction ranges from 0.07% to 2.4% [16]. The efficacy and safety of GBCAs were well confirmed during phase I-IV clinical trials [25–28]. The reports about the frequency of the appearance of AR are either after a single GBCA administration [18, 29–34] or without differentiation between an AR after a single application or multiple GBCA applications [21, 35, 36], or the authors selected only one examination per patient [37]. Nevertheless, the frequency of acute adverse reactions to GBCA is about eight times higher in patients with a previous reaction to GBCA, and guidelines have discussed the efficacy of premedication with corticosteroid and antihistamine for subsequent contrast-enhanced MRIs [16, 17]. AR can also occur, despite premedication with corticosteroids and antihistamines, as a so-called “breakthrough reaction” [38–41].

The purpose of this study was to determine the frequency and severity of adverse reactions to intravenously administered GBCAs in children based on the number of GBCA exposures.

## Materials and methods

The study was conducted in accordance with the Declaration of Helsinki, and approved by the Institutional Review Board of the Medical University of Vienna (IRB No. 1321/2018). Informed Consent Statement: Patient consent was waived due to the retrospective data analysis.

All pediatric patients of the Department of Pediatrics and Adolescent Medicine of the Medical University of Vienna, who underwent an abdominal MR scan in the Department of Biomedical Imaging and Image-guided Therapy between 2009 and 2020, were retrospectively reviewed by A.H., A.M., and A.He. from January 1^st^, 2021, to September 30^th^, 2023, with regard to the reported adverse reactions in their medical history. These were documented in a contrast agent questionnaire, by parents, internal radiology reports, or by referring doctors’ documents. In our department, all patients and parents fill out a contrast agent questionnaire to report previous adverse reactions to a contrast agent. They also must to wait 30 minutes after a contrast-enhanced MRI in our waiting room, before they can go home. In this way, we can identify immediate reactions, which are recorded in the radiology report. The patients and the parents are told to come back to our hospital if they observe any abnormality. They discuss the MRI findings with the referring doctor within five days after the MRI scan, and can also report any observed reaction after the MRI scan.

The reported AR and their verification and severity were evaluated and categorized according to the ESUR and ACR guideline classification system [16, 17]. Included were all patients with at least one GBCA-enhanced abdominal MR scan, and who were younger than 18 years of age at the time of the initial MR scan. Patients with only non-enhanced abdomen MR scans were excluded.

### Management of adverse reactions and work-up

In our pediatric center, the subsequent MRIs of patients with a suspected GBCA-associated adverse reactions are managed either by the institutional pediatric premedication regime, modified according to Greenberger et al. [16, 42, 43], or the adverse reactions are verified by an allergy work-up [43], after obtaining the permission of patients or parents if the patient is minor.

### Statistical analysis

The statistical analysis was performed using the software package SPSS, Version: 28.0.1.0 (IBM, Armonk, NY). Demographic data were analysed as absolute numbers, and distributions were presented as median and range. Variables were compared using a parametric (Student´s t-test) analysis. A Spearman ρ-correlation and a Chi-square test between the reported adverse reactions and the total number of GBCA applications, and the number of each applied GBCA was calculated. Results with a p-value <0.05 were considered significant.

## Results and discussion

**Fig 1:**
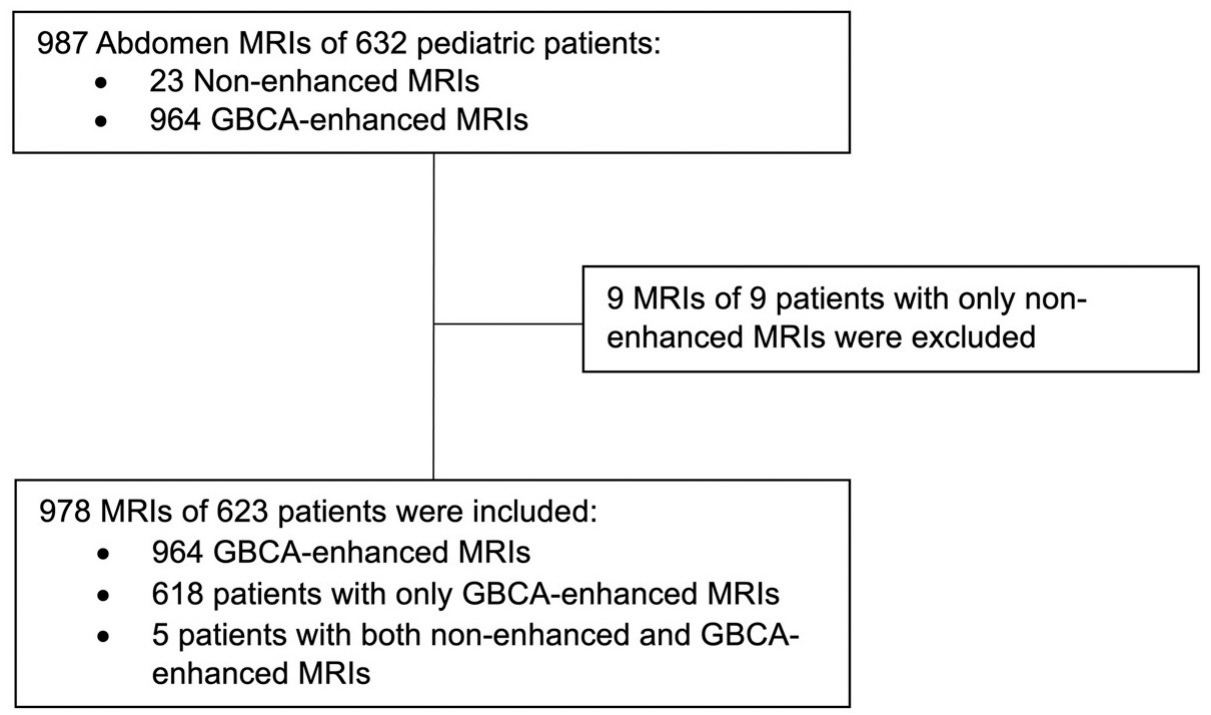
The selection process of the study population.

During the study period, 623 patients with 964 GBCA-enhanced abdomen MR scans were recorded. The selection process is summarized in Fig 1 and the Table 1 presents the demographic data of the study population.

**Table 1:** Study demographics.

Characters	Value
Number of Patients	623
Female	299 (48%)
Age at the first GBCA administration (y)	12.1 (0–17.9)
Age at the last GBCA administration (y)	14.4 (0–21.3)
Number of GBCA administrations per patient	1 (1–19)
Number of GBCA administrations in total	964
• Gadoxetate disodium (Primovist^®^)	626
• Gadoterate meglumine (Dotarem^®^)	247
• Gadobutrol (Gadovist^®^)	84
• Gadobenate dimeglumine (Multihance^®^)	6
• Gadoteridol (ProHance^®^)	1
Number of patients in each GBCA group	
• Gadoxetate disodium (Primovist^®^)	447
• Gadoterate meglumine (Dotarem^®^)	177
• Gadobutrol (Gadovist^®^)	67
• Gadobenate dimeglumine (Multihance^®^)	6
• Gadoteridol (ProHance^®^)	1
Number of GBCA administrations per patient	
• Gadoxetate disodium (Primovist^®^)	1 (1–14)
• Gadoterate meglumine (Dotarem^®^)	1 (1–12)
• Gadobutrol (Gadovist^®^)	1 (1–4)
• Gadobenate dimeglumine (Multihance^®^)	1 (0–1)
• Gadoteridol (ProHance^®^)	1 (0–1)
Number of patients with a single GBCA-enhanced MRI	464
• Gadoxetate disodium (Primovist^®^)	326
• Gadoterate meglumine (Dotarem^®^)	112
• Gadobutrol (Gadovist^®^)	25
• Gadobenate dimeglumine (Multihance^®^)	1
• Gadoteridol (ProHance^®^)	0
Number of patients with repeated GBCA-enhanced MRIs	159
Indications for MRIs	
• Abscess	12
• Appendicitis	32
• Congenital abdominal wall malformation	1
• GVHD	4
• Hepatobiliary functional disorders	216
• Intestinal bowel diseases	7
• Kidney and urological disorders	8
• Lymphoma	7
• Tumours	118
• Metabolic disorders	119
• Neurocutaneous disorders	12
• Pancreaticobiliary disorders	45
• PTLD	19
• Trauma	9
• Vascular abnormalities and disorders	14

Count variables are reported by count and relative frequency or the range in the parentheses.

GVHD: Graft versus host disease; PTLD: Post transplant lymphoma disease.

All contrast agent administration was performed based on clinical indications. For the administration of contrast agents, informed consent was obtained from the patients’ legal guardian or parents, respectively. Particularly in the case of Gd-EOB-DTPA administration, the legal guardian or parents were informed about the off-label use prior to administration of Gd-EOB-DTPA and their written consent was documented in the patient’s medical record.

One-hundred-fifty-nine patients had repeated GBCA-enhanced MR scans and five patients also had non-enhanced MR scans.

In the medical history of six patients, reports of suspected GBCA-associated adverse reactions were documented (Table 2).

**Table 2:** Clinical data of patients with a reported adverse reaction to a Gadolinium-based contrast agent.

Patient’s data during the study period					Reported adverse reactions in patient’s history											
Patient’s no	Patient’s age (y)	Sex	Diagnosis	No of GBCA-MRIs in total	Age at the first reported AR	GBCA at the first reported AR	Kind of reported AR	No of uneventful GBCA-MRIs before reported AR	Source of AR report				No of GBCA-MRIs with premedication	No of non-enhanced MRIs	Allergy work-up	GBCA-related AR could be ruled out
									CA questionnaire	Parents	Internal radiology reports	Referring Doctor				
1	8.3–16.2	F	NET	(16) 14 Gadoxetate disodium and 2 Gadoterate meglumine	14	Liver MRI with Gadoxetate disodium	Urticaria (mild)	12 Gadoxetate disodium and 1 Gadoterate meglumine	no	yes	no	yes	2 Gadoxetate disodium and 1 Gadoterate meglumine	1	no	no
2	7.5–13.5	M	ATRT -pancreatitis	(19) 13 Gadoxetate disodium and 6 Gadoterate meglumine	7	cMRI with Gadoterate meglumine	Urticaria (mild)	22 Gadoterate meglumine	yes	yes	no	yes	(19) 13 Gadoxetate disodium and 6 Gadoterate meglumine	9	no	no
3	14.1–16.2	F	LTX	(5) 2 Gadoxetate disodium and 3 Gadobutrol	11	unknown	unknown	unknown	no	no	no	Confused with IBCA	0	0	no	yes
4	6.6–13.0	M	LTX	6 Gadoxetate disodium	8	Gadoxetate disodium	Vomiting	3 Gadoxetate disodium	no	no	yes	no	0	0	no	no
5	13.6–16.5	F	IBD	5 Gadoterate meglumine	-	-	-	-	Confused with IBCA	yes	CT report	no	0	0	no	yes
6	13.7	F	Pancreatic mass	1 Gadobutrol	17	Gadobutrol	Urticaria	1	no	yes	no	no	0	0	no	yes

GBCA: Gadolinium-based contrast agent; AR: adverse reaction (both chemotoxic and allergy-like); CA: contrast agent; NET: neuroendocrine tumor;

ATRT: Atypical teratoid rhabdoid tumor; cMRI: cranial MRI; LTX: liver transplant; IBD: intestinal bowel disease; IBCA: iodine-based contrast agents;

CT: computed tomography

The reports of adverse reactions in three patients could be ruled out as GBCA-associated:

- Iodine-based contrast agent (IBCA)-associated AR during a computed tomography were recorded as a GBCA-associated AR on a GBCA-questionnaire sheet by parents of patient 5 and by referring doctors of patient 3. Patient 5 underwent five uneventful Gadoterate meglumine-enhanced MR scans and Patient 3 had five uneventful (three Gadoxetate disodium- and two Gadobutrol-enhanced) MR scans, without a premedication regime.
- The parents of patient 6, who had an atopic eczema, reported a Gadobutrol-associated AR on the outpatient questionnaire sheet five years after the only Gadobutrol-enhanced abdominal MRI, without any documented immediate AR, either on the related radiology report or in the patient’s medical history, as well as without any other MR scan during the last five years or GBCA-associated allergy work-up.

The reports of adverse reactions in three patients could not be ruled out, although, due to the parents’ refusal, none of these three patients underwent an allergy work-up.

- Patient 1 had urticaria that was categorized as a mild allergy-like reaction after the twelfth Gadoxetate disodium-enhanced MRI, but subsequent MRIs with the institutional pediatric premedication regime were uneventful.
- Patient 2 had urticaria that was categorized as a mild allergy-like reaction, after the twenty-second Gadoterate meglumine-enhanced MRI at the age of seven.
- Patient 4 had vomiting that was categorized as an immediate mild chemotoxic AR, after the fourth Gadoxetate disodium-enhanced MR scan. The subsequent Gadoxetate disodium-enhanced MRIs were uneventful without a premedication regime.

**Fig 2:**
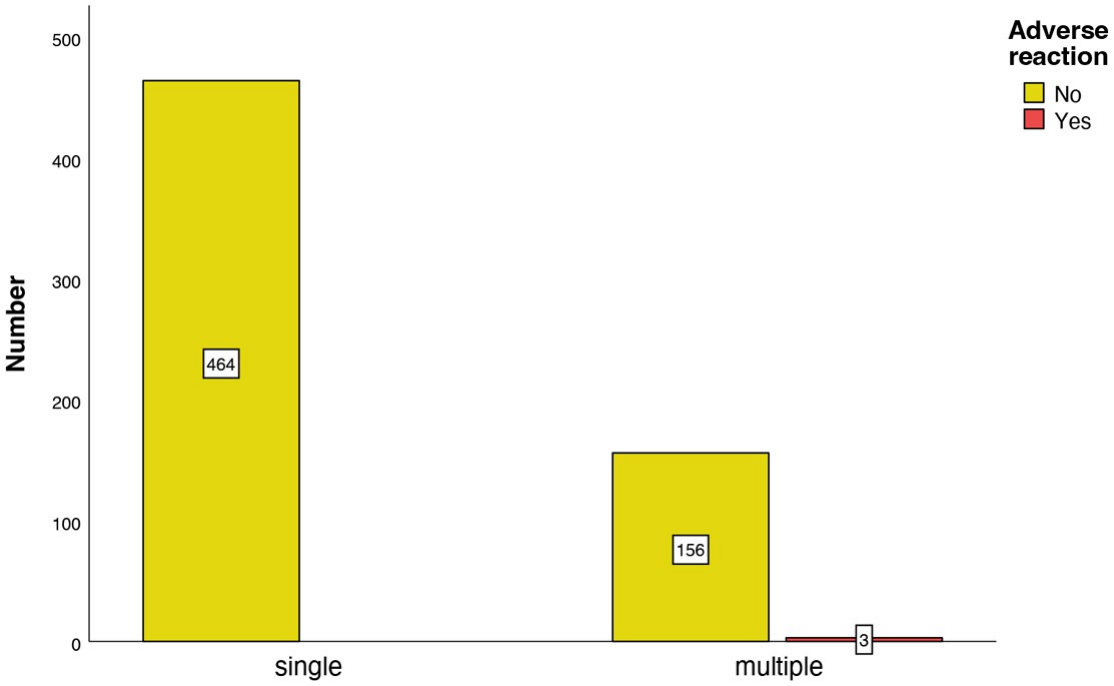
Number of adverse reactions after a single or after multiple GBCA-administration.

In 464 patients with only a single GBCA-enhanced MRI, there were no GBCA-associated AR (Fig 2).

The number of adverse reactions in patients who received one or more doses of the same GBCA are presented in Table 3.

**Table 3:** Adverse reactions in patients who received one or more doses of the same Gadolinium-based contrast agent.

Gadolinium-based contrast agent	Number of patients	Number and kind of adverse reactions
Gadoxetate disodium (Primovist^®^)	390	1 vomiting (mild chemotoxic)
Gadoterate meglumine (Dotarem^®^)	134	0
Gadobutrol (Gadovist^®^)	29	0
Gadobenate dimeglumine (Multihance^®^)	1	0
Gadoteridol (ProHance^®^)	1	0

In total, there were three reported AR of 964 GBCA doses administered in the whole study collective (3/964 = 0.31%), and all the reported AR were in the patient group with multiple GBCA administration (3/159 = 1.89%).

The Spearman ρ-correlation coefficient between the reported adverse reactions and the total number of GBCA applications and the number of each applied GBCA per patient are presented in Table 4.

**Table 4:** The Spearman ρ-correlation coefficient between the reported adverse reactions and the total number of GBCA applications and number of each GBCA application per patient.

	Total No of GBCA applications	No of Gadoxetate disodium applications	No of Gadoterate meglumine applications	No of Gadobutrol applications	No of Gadobenate dimeglumine applications	No of Gadoteridol applications
**Reported adverse reactions**	0.156 (<0.001)	0.210 (<0.001)	0.226 (0.002)	n.a.	n.a.	n.a.

The two-sided p-values are given in brackets (p<0.05 considered significant).

GBCA: Gadolinium-based contrast agent; n.a.: not applicable

A positive significant correlation was calculated between the reported adverse reactions and the total number of GBCA applications (p<0.001) and the number of Gadoxetate disodium (p<0.001) and Gadoterate meglumine (p = 0.002) applications.

The independent two-tailed t-test calculated a significant association between the reported adverse reactions and the total number of GBCA applications, in general (p<0.001), and the number of Gadoxetate disodium (p<0.001) and Gadoterate meglumine (p = 0.001), in particular.

The Chi-square test identified significantly higher adverse reactions reported in the patients who experienced repeated GBCA administrations compared to those with only one GBCA administration in the whole study population (p = 0.003), as well as in each GBCA group (for Gadoxetate disodium p<0.001; for Gadoterate meglumine (p<0.001). The t-test and the Chi-square test were not applicable for the few cases with Gadobenate dimeglumine (six cases) and Gadoteridol-enhanced MR scans (one case) in our study (Table 1). The tests were not applicable for Gadobutrol because of the lack of AR in this group, either for single or multiple applications.

In our pediatric study population, we observed that the reporting of GBCA-associated AR is higher in patients with multiple GBCA administrations and that the total number of GBCA applications correlated with the reported adverse reactions. Collectively, this observation reflects an increased individual risk of suffering an AR and an increased institutional risk of observing an AR with repeated GBCA investigations. No severe adverse reaction to GBCA were reported in our population. The suspected mild allergy-like (2/964 = 0.21%) and chemotoxic (1/964 = 0.10%) adverse reactions to GBCA in our study were rare. However, the frequency of suspected allergy-like and chemotoxic reactions in the patient group with multiple GBCA administrations was six times higher (2/159 = 1.26% resp. 1/159 = 0.63%).

Verified adverse reactions to a single exposure to GBCA were not observed in our patient groups.

Urticaria is, indeed, very commonly associated with infectious causes, especially in very young children [44]. Urticaria, as part of paraneoplastic syndromes, has also been described in children in the form of a) neurologic origin (opsoclonus-myoclonus, limbic, anti-N-methyl-d-aspartate [NMDA] and anti-Ma2 encephalitis and myasthenia gravis), b) endocrine origin (neuroendocrine tumors, hypercalcemia, SIADH [syndrome of inappropriate antidiuretic hormone secretion], osteomalacia/rickets, and ROHHAD [rapid onset of obesity, hypoventilation, hypothalamic dysfunction and autonomic dysregulation]), and c) dermatologic/rheumatologic origin (hypertrophic osteoarthropathy and paraneoplastic pemphigus) [45]. This possibility was assessed thoroughly in the history of the cases, but not detected.

Thus, we conclude that the manifestation of a drug-related reaction to GBCA nicely relates to the current view of drug hypersensitivity development. Repeated exposures and possibly time intervals between the exposures might be relevant inducing factors in addition to the intrinsic factors of the drug itself. Current knowledge of this process includes a time lag in which to form the hapten‐protein complex, to process the hapten‐protein, and to present the hapten‐peptides on HLA molecules, as well as for the expansion of drug‐specific T and B cells [46].

The published reports of GBCA-related AR after a single administration varied in the pediatric patients from 0% to 5.8%. Geller et al. reported no AR to Gadoxetate disodium-enhanced liver MRI in 52 pediatric patients [29], and Farmakis et al. observed, in a collective of 150 children younger than two years of age, two (1.3%) drug-related AR (wheezing and sneezing) after a single standard dose (0.1mmol/kg) of Gadoterate meglumine administration [30]. Chang et al. reported one case with vomiting after a single standard dose (0.1mmol/kg) of Gadoterate meglumine in 1631 children [31]. After a standard Gadobutrol dose (0.1 mmol/kg), Hahn et al. reported 5.8% drug-related AR in a collective of 130 pediatric patients younger than 18 years of age [32]. Bhargava et al. observed, in a collective of children younger than two years of age, no drug-related AR in 60 cases [33], but Kunze et al. reported 2.3% drug-related AR in 44 children of the same age group [34].

Reports of AR after multiple, initially well-tolerated GBCA administrations have not been published. In general, the published reports of GBCA safety stated the rate of AR in large patient populations without any discussion about the number of GBCA administrations before the initial adverse reaction in each individual [21, 22, 25, 35, 36, 47]. Kim et al. also included only one examination per patient either the scan in which an acute adverse reaction was occurred, or the most recent scan, if there was no adverse reaction recorded [37]. Taking into account that the likelihood of the occurrence of an adverse reaction is higher in patients with a previous reaction in their medical history [16, 17], it is particularly crucial in oncological patients, to identify the tolerable GBCA for subsequent MR scans [17, 48]. Due to the appearance of “breakthrough reactions” despite premedication with corticosteroid and antihistamine [39, 43, 49], an expert allergy consult is required to evaluate the evidence of true allergy to the contrast agent and the evidence of cross-reactivity to other contrast agents. In this way, GBCA-associated adverse reactions can be better anticipated and managed for subsequent MR scans [17, 43, 50].

Nevertheless, we could suggest that a reported AR associated with a GBCA administration, either by the parents or the patient, or by referral doctors, should be carefully evaluated, because confusion with IBCA (patients 3 and 5) or the coexistence of atopic diseases (patient 6) could be misinterpreted as an AR. An immediate reaction after GBCA administration such as vomiting, could be interpreted either as chemotoxic or as a reaction due to some other condition such as hunger (patient 4). Finally, in our study population, none of the reported AR were confirmed by an allergy work-up, either because of parents’ refusal (patients 1 and 2), or because of the radiologists’ assessment of the association of GBCA with the reported adverse reaction (patient 4).

### Limitations

1. Due to the retrospective character of our study, the reported adverse reactions could not be verified, specifically, by allergy-testing.
2. Due to the small study collective and due to the size difference of the two patient groups–with and without reported adverse reactions–the effect of the total number of applied GBCAs, and the number of each GBCA on the reported GBCA-associated adverse reactions could not be evaluated, e.g., a binary logistic regression analysis.
3. It should also be noted that the study compared different contrast agent combinations, which limits the conclusions about individual contrast agents.
4. A prospective multicentre study design would allow more reliable conclusions to be drawn.

## Conclusion

GBCA-associated adverse reactions are very rare in children. But the reporting of GBCA-associated adverse reactions is higher in patients with multiple GBCA administrations and the total number of GBCA applications significantly correlated with the reported adverse reactions. Not all AR reports are GBCA-associated, but all AR reports should be carefully evaluated and confirmed, due to an increasing likelihood of drug hypersensitivity upon repeated drug exposures.

## Supporting information

S1 File Data. (PDF)

## Abbreviations

AR: Adverse reactions
ATRT: Atypical teratoid rhabdoid tumor
CA: Contrast agent
cMRI: Cranial magnetic resonance imaging
CT: Computed tomography
GBCA: Gadolinium-based contrast agent
GVHD: Graft versus host disease
IBCA: Iodine-based contrast agents
IBD: Intestinal bowel disease
LTX: Liver transplant
MRI: Magnetic resonance imaging
NET: Neuroendocrine tumor
PTLD: Post transplant lymphoma disease
