# Supplementary material for: Acute adverse reactions after multiple initially well-tolerated gadolinium-based contrast-enhanced abdomen MRIs in pediatric patients
Source: PLoS One. 2024 Dec 3;19(12):e0313495. doi: 10.1371/journal.pone.0313495 (PMC11614221; doi:10.1371/journal.pone.0313495)
Supplement: S1 File — (PDF) [file pone.0313495.s001.pdf]

Data

Patient's no	No of unenhanced MRIs	Gadoxetate disodium	Gadoterate meglumine	Gadobutrol	Gadobenate dimeglumine	Gadoteridol	Total No of GBCA administrations	Reported adverse reaction (yes=1. no=0)	Suspected adverse reaction (yes=1. no=0)	Indication for MRI
**1**	1	14	2				16	1	1	Tumour
**2**	9	13	6				19	1	1	Pancreaticobiliary disorder
**3**		2		3			5	1	0	PTLD
**4**		6					6	1	1	PTLD
**5**			5				5	1	0	IBD
**6**				1			1	1	0	Tumour
**7**		3					3	0	0	Metabolic disorder
**8**		2					2	0	0	Metabolic disorder
**9**			4				4	0	0	Tumour
**10**		2	3				5	0	0	Hepatobiliary functional disorder
**11**		1					1	0	0	Tumour
**12**		1					1	0	0	Pancreaticobiliary disorder
**13**				1			1	0	0	Lymphoma
**14**		1					1	0	0	Hepatobiliary functional disorder
**15**			1				1	0	0	Vascular abnormalities and disorder
**16**			2				2	0	0	Hepatobiliary functional disorder
**17**		1					1	0	0	Hepatobiliary functional disorder
**18**		1					1	0	0	Pancreaticobiliary disorder
**19**			1	2			3	0	0	Neurocutaneous disorder
**20**		1					1	0	0	Tumour
**21**			1				1	0	0	Hepatobiliary functional disorder
**22**		1					1	0	0	Hepatobiliary functional disorder
**23**			1	2	1		4	0	0	Neurocutaneous disorder
**24**		1					1	0	0	Metabolic disorder
**25**		1					1	0	0	Pancreaticobiliary disorder
**26**			1				1	0	0	Tumour
**27**		1					1	0	0	Metabolic disorder
**28**				1			1	0	0	Appendicitis
**29**			1				1	0	0	Tumour
**30**		1					1	0	0	Metabolic disorder
**31**		1					1	0	0	Hepatobiliary functional disorder
**32**		1					1	0	0	Hepatobiliary functional disorder
**33**		1					1	0	0	Hepatobiliary functional disorder
**34**			1				1	0	0	Tumour
**35**			1				1	0	0	Lymphoma
**36**		1					1	0	0	Hepatobiliary functional disorder
**37**			6	2			8	0	0	Neurocutaneous disorder
**38**		1		1			2	0	0	Hepatobiliary functional disorder
**39**		1					1	0	0	Hepatobiliary functional disorder
**40**		1		1			2	0	0	Hepatobiliary functional disorder
**41**		1					1	0	0	Metabolic disorder
**42**			1				1	0	0	Tumour
**43**		1					1	0	0	Hepatobiliary functional disorder
**44**		1					1	0	0	Hepatobiliary functional disorder
**45**		1					1	0	0	Tumour
**46**		1					1	0	0	Hepatobiliary functional disorder
**47**		1					1	0	0	Hepatobiliary functional disorder

**48**		1					1	0	0	Hepatobiliary functional disorder
**49**		1					1	0	0	Hepatobiliary functional disorder
**50**		1					1	0	0	Pancreaticobiliary disorder
**51**				1			1	0	0	Appendicitis
**52**		1					1	0	0	Hepatobiliary functional disorder
**53**			1				1	0	0	Hepatobiliary functional disorder
**54**		1					1	0	0	Hepatobiliary functional disorder
**55**		1					1	0	0	Trauma
**56**			1				1	0	0	Abscess
**57**		2					2	0	0	Pancreaticobiliary disorder
**58**		1		1			2	0	0	Hepatobiliary functional disorder
**59**		1					1	0	0	Metabolic disorder
**60**		1					1	0	0	Metabolic disorder
**61**		1					1	0	0	Tumour
**62**		1					1	0	0	Metabolic disorder
**63**				4			4	0	0	Abscess
**64**			2				2	0	0	Neurocutaneous disorder
**65**		1					1	0	0	Metabolic disorder
**66**		1					1	0	0	Hepatobiliary functional disorder
**67**		2					2	0	0	Metabolic disorder
**68**		1					1	0	0	Metabolic disorder
**69**		1					1	0	0	Hepatobiliary functional disorder
**70**		1					1	0	0	Metabolic disorder
**71**				2			2	0	0	Kidney and urological disorder
**72**		1					1	0	0	Metabolic disorder
**73**		6					6	0	0	PTLD
**74**			1				1	0	0	Lymphoma
**75**		2					2	0	0	Hepatobiliary functional disorder
**76**		1					1	0	0	Hepatobiliary functional disorder
**77**		1					1	0	0	Hepatobiliary functional disorder
**78**			1				1	0	0	Tumour
**79**		1					1	0	0	Metabolic disorder
**80**		1					1	0	0	Tumour
**81**		1		1			2	0	0	Hepatobiliary functional disorder
**82**			1				1	0	0	Vascular abnormalities and disorder
**83**		1	1	1			3	0	0	Hepatobiliary functional disorder
**84**			1				1	0	0	Vascular abnormalities and disorder
**85**			1		1		2	0	0	Hepatobiliary functional disorder
**86**				1			1	0	0	Pancreaticobiliary disorder
**87**		1					1	0	0	Metabolic disorder
**88**		1					1	0	0	Metabolic disorder
**89**			4				4	0	0	Hepatobiliary functional disorder
**90**		1					1	0	0	Metabolic disorder
**91**		1					1	0	0	Hepatobiliary functional disorder
**92**		1					1	0	0	Tumour
**93**		1					1	0	0	Metabolic disorder
**94**		4					4	0	0	Hepatobiliary functional disorder
**95**		1					1	0	0	Hepatobiliary functional disorder
**96**		1					1	0	0	Hepatobiliary functional disorder
**97**		1					1	0	0	Metabolic disorder

**98**		1					1	0	0	Hepatobiliary functional disorder
**99**		1					1	0	0	Metabolic disorder
**100**		1	1				2	0	0	Metabolic disorder
**101**		1					1	0	0	Metabolic disorder
**102**				1			1	0	0	CED
**103**			1				1	0	0	Tumour
**104**			1				1	0	0	Tumour
**105**			1				1	0	0	Appendicitis
**106**		1					1	0	0	GVHD
**107**		1					1	0	0	Metabolic disorder
**108**		1					1	0	0	Hepatobiliary functional disorder
**109**		1					1	0	0	Hepatobiliary functional disorder
**110**		1					1	0	0	Hepatobiliary functional disorder
**111**			1				1	0	0	Kidney and urological disorder
**112**		3		1			4	0	0	Pancreaticobiliary disorder
**113**		3		2			5	0	0	Pancreaticobiliary disorder
**114**			1				1	0	0	Tumour
**115**		1					1	0	0	Metabolic disorder
**116**			1	1	1		3	0	0	CED
**117**			1				1	0	0	Appendicitis
**118**		1					1	0	0	Metabolic disorder
**119**		1					1	0	0	Pancreaticobiliary disorder
**120**		1					1	0	0	Metabolic disorder
**121**		1					1	0	0	Metabolic disorder
**122**			1				1	0	0	Pancreaticobiliary disorder
**123**		1					1	0	0	Pancreaticobiliary disorder
**124**		1					1	0	0	Hepatobiliary functional disorder
**125**		1					1	0	0	Metabolic disorder
**126**		1					1	0	0	Metabolic disorder
**127**		3					3	0	0	Pancreaticobiliary disorder
**128**		1	3				4	0	0	Hepatobiliary functional disorder
**129**		2					2	0	0	Pancreaticobiliary disorder
**130**		1					1	0	0	Metabolic disorder
**131**		1					1	0	0	Hepatobiliary functional disorder
**132**		1					1	0	0	GVHD
**133**			2				2	0	0	GVHD
**134**			1				1	0	0	Appendicitis
**135**			1				1	0	0	Vascular abnormalities and disorder
**136**			2	1			3	0	0	Tumour
**137**			1				1	0	0	Pancreaticobiliary disorder
**138**		1					1	0	0	Metabolic disorder
**139**				1			1	0	0	Tumour
**140**		1					1	0	0	Metabolic disorder
**141**			1				1	0	0	Tumour
**142**		1	2				3	0	0	Tumour
**143**			1				1	0	0	Appendicitis
**144**		1					1	0	0	Metabolic disorder
**145**			1				1	0	0	Vascular abnormalities and disorder
**146**		1					1	0	0	Hepatobiliary functional disorder
**147**		1					1	0	0	Metabolic disorder

**148**		1					1	0	0	Metabolic disorder
**149**		1					1	0	0	Tumour
**150**				1			1	0	0	Appendicitis
**151**			1				1	0	0	Appendicitis
**152**		2					2	0	0	Hepatobiliary functional disorder
**153**		1					1	0	0	Hepatobiliary functional disorder
**154**		5					5	0	0	Tumour
**155**		1					1	0	0	Hepatobiliary functional disorder
**156**		2					2	0	0	Tumour
**157**			1				1	0	0	Vascular abnormalities and disorder
**158**				1			1	0	0	CED
**159**		2					2	0	0	Hepatobiliary functional disorder
**160**		1		1			2	0	0	Pancreaticobiliary disorder
**161**			1				1	0	0	Hepatobiliary functional disorder
**162**		4					4	0	0	Hepatobiliary functional disorder
**163**		1					1	0	0	Metabolic disorder
**164**		1					1	0	0	GVHD
**165**			12	3			15	0	0	Tumour
**166**		1					1	0	0	Tumour
**167**			1				1	0	0	Kidney and urological disorder
**168**			1				1	0	0	Abscess
**169**		1					1	0	0	PTLD
**170**		2		1			3	0	0	Hepatobiliary functional disorder
**171**			1				1	0	0	Tumour
**172**		1					1	0	0	Hepatobiliary functional disorder
**173**				1			1	0	0	Appendicitis
**174**		1					1	0	0	Hepatobiliary functional disorder
**175**		2					2	0	0	Hepatobiliary functional disorder
**176**			1				1	0	0	Tumour
**177**		1					1	0	0	Hepatobiliary functional disorder
**178**		1					1	0	0	Hepatobiliary functional disorder
**179**		1					1	0	0	Hepatobiliary functional disorder
**180**		1					1	0	0	Hepatobiliary functional disorder
**181**			1				1	0	0	Appendicitis
**182**		1					1	0	0	Hepatobiliary functional disorder
**183**			1				1	0	0	Tumour
**184**		1					1	0	0	Hepatobiliary functional disorder
**185**		1					1	0	0	Hepatobiliary functional disorder
**186**		1					1	0	0	Appendicitis
**187**			1				1	0	0	PTLD
**188**			1				1	0	0	Tumour
**189**		1					1	0	0	Hepatobiliary functional disorder
**190**		1					1	0	0	Metabolic disorder
**191**			1				1	0	0	Tumour
**192**		1	2				3	0	0	PTLD
**193**		1					1	0	0	Metabolic disorder
**194**		1					1	0	0	Hepatobiliary functional disorder
**195**		1					1	0	0	PTLD
**196**		6	4				10	0	0	PTLD
**197**		2					2	0	0	Metabolic disorder

**198**		1					1	0	0	Hepatobiliary functional disorder
**199**		1					1	0	0	Hepatobiliary functional disorder
**200**		1					1	0	0	Pancreaticobiliary disorder
**201**		1					1	0	0	Metabolic disorder
**202**		2		1			3	0	0	trauma
**203**			1				1	0	0	Tumour
**204**		1					1	0	0	Metabolic disorder
**205**			1				1	0	0	Tumour
**206**			1				1	0	0	Appendicitis
**207**		1					1	0	0	Metabolic disorder
**208**		1					1	0	0	PTLD
**209**			2				2	0	0	Neurocutaneous disorder
**210**		1					1	0	0	Hepatobiliary functional disorder
**211**			1				1	0	0	Appendicitis
**212**		3					3	0	0	Hepatobiliary functional disorder
**213**		1		1			2	0	0	Hepatobiliary functional disorder
**214**			1				1	0	0	Tumour
**215**		1		1			2	0	0	Tumour
**216**		1					1	0	0	Hepatobiliary functional disorder
**217**		1					1	0	0	Hepatobiliary functional disorder
**218**		1					1	0	0	Pancreaticobiliary disorder
**219**		1					1	0	0	Metabolic disorder
**220**			1				1	0	0	Tumour
**221**		1					1	0	0	Hepatobiliary functional disorder
**222**		1	1				2	0	0	Tumour
**223**		2					2	0	0	Hepatobiliary functional disorder
**224**			1				1	0	0	Tumour
**225**		1					1	0	0	Tumour
**226**		1					1	0	0	Metabolic disorder
**227**			1				1	0	0	Abscess
**228**		1		1			2	0	0	Hepatobiliary functional disorder
**229**		1		1			2	0	0	Hepatobiliary functional disorder
**230**		5					5	0	0	Tumour
**231**		1					1	0	0	Hepatobiliary functional disorder
**232**			1				1	0	0	Tumour
**233**		1					1	0	0	Hepatobiliary functional disorder
**234**		1		2			3	0	0	Hepatobiliary functional disorder
**235**		1	1				2	0	0	Tumour
**236**		1					1	0	0	trauma
**237**		1					1	0	0	Pancreaticobiliary disorder
**238**		1					1	0	0	Metabolic disorder
**239**		1					1	0	0	Metabolic disorder
**240**		1					1	0	0	Hepatobiliary functional disorder
**241**		1					1	0	0	Hepatobiliary functional disorder
**242**			1				1	0	0	Tumour
**243**			1				1	0	0	Abscess
**244**		1					1	0	0	Metabolic disorder
**245**		5	1				6	0	0	Hepatobiliary functional disorder
**246**			1				1	0	0	Tumour
**247**			1				1	0	0	Tumour

**248**		1					1	0	0	Tumour
**249**			1				1	0	0	Appendicitis
**250**		1					1	0	0	Metabolic disorder
**251**		1					1	0	0	Hepatobiliary functional disorder
**252**		1					1	0	0	Metabolic disorder
**253**			1				1	0	0	Tumour
**254**				1			1	0	0	Appendicitis
**255**		1					1	0	0	Hepatobiliary functional disorder
**256**		1					1	0	0	Metabolic disorder
**257**		1					1	0	0	Appendicitis
**258**		1					1	0	0	Metabolic disorder
**259**		1					1	0	0	Metabolic disorder
**260**		1					1	0	0	Metabolic disorder
**261**		4	1				5	0	0	PTLD
**262**		1					1	0	0	Metabolic disorder
**263**		2					2	0	0	Pancreaticobiliary disorder
**264**		1					1	0	0	Hepatobiliary functional disorder
**265**		1					1	0	0	Hepatobiliary functional disorder
**266**			1				1	0	0	trauma
**267**		1					1	0	0	Lymphoma
**268**		2					2	0	0	Tumour
**269**		1					1	0	0	Metabolic disorder
**270**			1				1	0	0	Neurocutaneous disorder
**271**		1					1	0	0	Metabolic disorder
**272**		2					2	0	0	Tumour
**273**		1	1				2	0	0	Hepatobiliary functional disorder
**274**			1				1	0	0	Neurocutaneous disorder
**275**		1	1				2	0	0	Pancreaticobiliary disorder
**276**		1					1	0	0	Pancreaticobiliary disorder
**277**		1					1	0	0	Metabolic disorder
**278**		1		1			2	0	0	Hepatobiliary functional disorder
**279**		1					1	0	0	Metabolic disorder
**280**			1				1	0	0	Appendicitis
**281**		1		1			2	0	0	Tumour
**282**			1				1	0	0	trauma
**283**		3					3	0	0	Hepatobiliary functional disorder
**284**			1				1	0	0	Appendicitis
**285**		1					1	0	0	Metabolic disorder
**286**			1				1	0	0	Pancreaticobiliary disorder
**287**			1				1	0	0	Tumour
**288**		1					1	0	0	Hepatobiliary functional disorder
**289**		1					1	0	0	Metabolic disorder
**290**		2					2	0	0	Hepatobiliary functional disorder
**291**		1					1	0	0	Hepatobiliary functional disorder
**292**		2					2	0	0	Hepatobiliary functional disorder
**293**		1					1	0	0	Hepatobiliary functional disorder
**294**			1				1	0	0	Tumour
**295**		1					1	0	0	Hepatobiliary functional disorder
**296**			1				1	0	0	Vascular abnormalities and disorder
**297**		2					2	0	0	Hepatobiliary functional disorder

**298**		1					1	0	0	Pancreaticobiliary disorder
**299**		1					1	0	0	Metabolic disorder
**300**		1					1	0	0	Hepatobiliary functional disorder
**301**		1	1				2	0	0	Hepatobiliary functional disorder
**302**		1					1	0	0	Hepatobiliary functional disorder
**303**		1					1	0	0	Hepatobiliary functional disorder
**304**		1					1	0	0	Hepatobiliary functional disorder
**305**		1					1	0	0	Hepatobiliary functional disorder
**306**		2	1				3	0	0	Hepatobiliary functional disorder
**307**		1	1				2	0	0	Tumour
**308**		1		1			2	0	0	Tumour
**309**		1					1	0	0	Metabolic disorder
**310**		1					1	0	0	Hepatobiliary functional disorder
**311**			2				2	0	0	Tumour
**312**			1				1	0	0	Tumour
**313**		1					1	0	0	Metabolic disorder
**314**			1				1	0	0	Lymphoma
**315**			1				1	0	0	Appendicitis
**316**		1	1				2	0	0	Hepatobiliary functional disorder
**317**		2					2	0	0	Hepatobiliary functional disorder
**318**		1					1	0	0	Hepatobiliary functional disorder
**319**		1					1	0	0	Hepatobiliary functional disorder
**320**		1					1	0	0	Hepatobiliary functional disorder
**321**			1				1	0	0	Tumour
**322**		3					3	0	0	Abscess
**323**		1					1	0	0	PTLD
**324**		1					1	0	0	Metabolic disorder
**325**		1					1	0	0	Hepatobiliary functional disorder
**326**		2					2	0	0	Hepatobiliary functional disorder
**327**			1				1	0	0	Tumour
**328**				1			1	0	0	Appendicitis
**329**		1					1	0	0	Hepatobiliary functional disorder
**330**			1				1	0	0	Appendicitis
**331**		1					1	0	0	Metabolic disorder
**332**		1					1	0	0	Abscess
**333**		1					1	0	0	Metabolic disorder
**334**		1					1	0	0	Metabolic disorder
**335**		1					1	0	0	Pancreaticobiliary disorder
**336**		1					1	0	0	Hepatobiliary functional disorder
**337**		1					1	0	0	Hepatobiliary functional disorder
**338**			1				1	0	0	Abscess
**339**		2					2	0	0	Hepatobiliary functional disorder
**340**		3					3	0	0	PTLD
**341**		1					1	0	0	Hepatobiliary functional disorder
**342**			1				1	0	0	Kidney and urological disorder
**343**				1			1	0	0	Tumour
**344**		1					1	0	0	Metabolic disorder
**345**		1		1			2	0	0	Hepatobiliary functional disorder
**346**		2					2	0	0	Metabolic disorder
**347**		1					1	0	0	trauma

**348**		1					1	0	0	Hepatobiliary functional disorder
**349**		1					1	0	0	Pancreaticobiliary disorder
**350**		1					1	0	0	Metabolic disorder
**351**		1					1	0	0	Hepatobiliary functional disorder
**352**		3					3	0	0	trauma
**353**		1					1	0	0	Metabolic disorder
**354**		1					1	0	0	Hepatobiliary functional disorder
**355**		1					1	0	0	Metabolic disorder
**356**		2	1				3	0	0	Hepatobiliary functional disorder
**357**		1					1	0	0	Hepatobiliary functional disorder
**358**		1	1		1		3	0	0	Tumour
**359**		1					1	0	0	Hepatobiliary functional disorder
**360**	1		3				3	0	0	Tumour
**361**		1					1	0	0	Hepatobiliary functional disorder
**362**			2				2	0	0	Lymphoma
**363**			1				1	0	0	Abscess
**364**			1				1	0	0	Appendicitis
**365**			1				1	0	0	Tumour
**366**			1				1	0	0	Tumour
**367**		5					5	0	0	Hepatobiliary functional disorder
**368**		1		1			2	0	0	Hepatobiliary functional disorder
**369**		1					1	0	0	Metabolic disorder
**370**		2					2	0	0	Pancreaticobiliary disorder
**371**			1				1	0	0	CED
**372**		1					1	0	0	Hepatobiliary functional disorder
**373**		1					1	0	0	Metabolic disorder
**374**	1	4					4	0	0	Pancreaticobiliary disorder
**375**			1				1	0	0	Tumour
**376**		1					1	0	0	Metabolic disorder
**377**		1					1	0	0	Hepatobiliary functional disorder
**378**		1					1	0	0	Tumour
**379**		1					1	0	0	Metabolic disorder
**380**		4					4	0	0	Pancreaticobiliary disorder
**381**		1					1	0	0	Metabolic disorder
**382**		1					1	0	0	Hepatobiliary functional disorder
**383**		1					1	0	0	Tumour
**384**		1					1	0	0	Tumour
**385**			1				1	0	0	Vascular abnormalities and disorder
**386**			1				1	0	0	CED
**387**			1				1	0	0	Tumour
**388**			1				1	0	0	Congenital abdominal wall malformation
**389**		1					1	0	0	Tumour
**390**				1			1	0	0	Appendicitis
**391**		1					1	0	0	Hepatobiliary functional disorder
**392**		1					1	0	0	Pancreaticobiliary disorder
**393**		1					1	0	0	Hepatobiliary functional disorder
**394**		3					3	0	0	Tumour
**395**		1					1	0	0	Hepatobiliary functional disorder
**396**		1	1				2	0	0	Hepatobiliary functional disorder

**397**		1	3				4	0	0	Vascular abnormalities and disorder
**398**		1					1	0	0	Hepatobiliary functional disorder
**399**		3					3	0	0	Tumour
**400**		2					2	0	0	Pancreaticobiliary disorder
**401**				1			1	0	0	Tumour
**402**			1				1	0	0	CED
**403**		2					2	0	0	Hepatobiliary functional disorder
**404**		1					1	0	0	Tumour
**405**		2					2	0	0	Tumour
**406**		2					2	0	0	PTLD
**407**		1					1	0	0	PTLD
**408**		6					6	0	0	Tumour
**409**		1					1	0	0	Metabolic disorder
**410**		1					1	0	0	Hepatobiliary functional disorder
**411**		1					1	0	0	Metabolic disorder
**412**			3				3	0	0	Tumour
**413**		1					1	0	0	Tumour
**414**			1				1	0	0	Tumour
**415**		1					1	0	0	Hepatobiliary functional disorder
**416**		1					1	0	0	Hepatobiliary functional disorder
**417**		2	1				3	0	0	Tumour
**418**		1					1	0	0	Metabolic disorder
**419**			1				1	0	0	Tumour
**420**		1					1	0	0	Metabolic disorder
**421**		1					1	0	0	Hepatobiliary functional disorder
**422**			1				1	0	0	Appendicitis
**423**		4	2				6	0	0	Hepatobiliary functional disorder
**424**		1					1	0	0	Hepatobiliary functional disorder
**425**			2				2	0	0	Abscess
**426**					1		1	0	0	Tumour
**427**		1					1	0	0	Metabolic disorder
**428**		1					1	0	0	Hepatobiliary functional disorder
**429**		5					5	0	0	PTLD
**430**				1			1	0	0	Tumour
**431**			1				1	0	0	Appendicitis
**432**			1				1	0	0	Kidney and urological disorder
**433**		1					1	0	0	Pancreaticobiliary disorder
**434**			1				1	0	0	Tumour
**435**			1				1	0	0	Tumour
**436**				1			1	0	0	Appendicitis
**437**		1					1	0	0	PTLD
**438**		2					2	0	0	Hepatobiliary functional disorder
**439**		1					1	0	0	Hepatobiliary functional disorder
**440**	1	1					1	0	0	Hepatobiliary functional disorder
**441**			1				1	0	0	Hepatobiliary functional disorder
**442**			3			1	4	0	0	Neurocutaneous disorder
**443**		1					1	0	0	Hepatobiliary functional disorder
**444**			2				2	0	0	Vascular abnormalities and disorder
**445**		1	1				2	0	0	Hepatobiliary functional disorder
**446**		1					1	0	0	Metabolic disorder

**447**		1					1	0	0	Hepatobiliary functional disorder
**448**		1		1			2	0	0	Hepatobiliary functional disorder
**449**		1					1	0	0	Tumour
**450**			1				1	0	0	Hepatobiliary functional disorder
**451**			1				1	0	0	Tumour
**452**		1					1	0	0	Hepatobiliary functional disorder
**453**		1					1	0	0	Metabolic disorder
**454**				1			1	0	0	Abscess
**455**		1					1	0	0	Hepatobiliary functional disorder
**456**		1					1	0	0	Hepatobiliary functional disorder
**457**			2				2	0	0	Appendicitis
**458**		1					1	0	0	Hepatobiliary functional disorder
**459**		1					1	0	0	Hepatobiliary functional disorder
**460**		1					1	0	0	Hepatobiliary functional disorder
**461**		1	1	1			3	0	0	Hepatobiliary functional disorder
**462**				1			1	0	0	Vascular abnormalities and disorder
**463**		1					1	0	0	Pancreaticobiliary disorder
**464**		1					1	0	0	Hepatobiliary functional disorder
**465**				1			1	0	0	Tumour
**466**		1					1	0	0	Metabolic disorder
**467**		2					2	0	0	Metabolic disorder
**468**		1					1	0	0	Hepatobiliary functional disorder
**469**		1	1				2	0	0	Hepatobiliary functional disorder
**470**		1					1	0	0	Metabolic disorder
**471**		1					1	0	0	Metabolic disorder
**472**		2					2	0	0	Hepatobiliary functional disorder
**473**			1				1	0	0	Pancreaticobiliary disorder
**474**		1					1	0	0	Tumour
**475**				1			1	0	0	Appendicitis
**476**		1					1	0	0	Hepatobiliary functional disorder
**477**		1					1	0	0	Hepatobiliary functional disorder
**478**		1					1	0	0	Hepatobiliary functional disorder
**479**		1					1	0	0	Tumour
**480**		5					5	0	0	Tumour
**481**		1					1	0	0	Metabolic disorder
**482**			3				3	0	0	PTLD
**483**		1	1				2	0	0	Tumour
**484**		1					1	0	0	Hepatobiliary functional disorder
**485**			1				1	0	0	Tumour
**486**		1					1	0	0	Tumour
**487**		1					1	0	0	Metabolic disorder
**488**			3				3	0	0	Hepatobiliary functional disorder
**489**		1					1	0	0	Hepatobiliary functional disorder
**490**		3					3	0	0	Hepatobiliary functional disorder
**491**		1					1	0	0	Metabolic disorder
**492**		1		1			2	0	0	Hepatobiliary functional disorder
**493**			1				1	0	0	Tumour
**494**		1					1	0	0	Abscess
**495**			1				1	0	0	Tumour
**496**		1					1	0	0	Metabolic disorder

**497**		4					4	0	0	Hepatobiliary functional disorder
**498**			1				1	0	0	Tumour
**499**		2					2	0	0	Tumour
**500**		1					1	0	0	Hepatobiliary functional disorder
**501**			1				1	0	0	Tumour
**502**		1					1	0	0	PTLD
**503**		1					1	0	0	Hepatobiliary functional disorder
**504**		1					1	0	0	Hepatobiliary functional disorder
**505**		3					3	0	0	Hepatobiliary functional disorder
**506**		1					1	0	0	Metabolic disorder
**507**			2				2	0	0	Tumour
**508**		1		1			2	0	0	Hepatobiliary functional disorder
**509**		1					1	0	0	Metabolic disorder
**510**		1					1	0	0	Hepatobiliary functional disorder
**511**		1					1	0	0	Metabolic disorder
**512**		2					2	0	0	Hepatobiliary functional disorder
**513**				1			1	0	0	Tumour
**514**			1				1	0	0	Tumour
**515**		1					1	0	0	Hepatobiliary functional disorder
**516**		2					2	0	0	trauma
**517**		2		1			3	0	0	Hepatobiliary functional disorder
**518**		1					1	0	0	Pancreaticobiliary disorder
**519**			1				1	0	0	Tumour
**520**		1					1	0	0	Hepatobiliary functional disorder
**521**		1					1	0	0	Hepatobiliary functional disorder
**522**		1					1	0	0	Hepatobiliary functional disorder
**523**		1					1	0	0	Metabolic disorder
**524**		1					1	0	0	Hepatobiliary functional disorder
**525**		1					1	0	0	Metabolic disorder
**526**		1					1	0	0	Hepatobiliary functional disorder
**527**		5	1				6	0	0	Hepatobiliary functional disorder
**528**			1	1			2	0	0	Tumour
**529**		1					1	0	0	Hepatobiliary functional disorder
**530**		1					1	0	0	Metabolic disorder
**531**		1					1	0	0	Metabolic disorder
**532**			1	2			3	0	0	Hepatobiliary functional disorder
**533**			1				1	0	0	Kidney and urological disorder
**534**				1			1	0	0	Tumour
**535**			1				1	0	0	Tumour
**536**			2				2	0	0	Tumour
**537**		1					1	0	0	Metabolic disorder
**538**		1					1	0	0	Tumour
**539**		1					1	0	0	Hepatobiliary functional disorder
**540**		6					6	0	0	trauma
**541**		1					1	0	0	Hepatobiliary functional disorder
**542**		1					1	0	0	Hepatobiliary functional disorder
**543**		1					1	0	0	Tumour
**544**		2					2	0	0	Tumour
**545**		1					1	0	0	Pancreaticobiliary disorder
**546**		1					1	0	0	Metabolic disorder

**547**		1					1	0	0	Pancreaticobiliary disorder
**548**		1					1	0	0	Hepatobiliary functional disorder
**549**		4					4	0	0	Hepatobiliary functional disorder
**550**		2					2	0	0	Metabolic disorder
**551**				1			1	0	0	Appendicitis
**552**		1					1	0	0	Tumour
**553**		3					3	0	0	PTLD
**554**		1					1	0	0	Metabolic disorder
**555**			1				1	0	0	Kidney and urological disorder
**556**		1					1	0	0	Metabolic disorder
**557**			1				1	0	0	Pancreaticobiliary disorder
**558**		1					1	0	0	Hepatobiliary functional disorder
**559**		1					1	0	0	Hepatobiliary functional disorder
**560**			1				1	0	0	Appendicitis
**561**		1					1	0	0	Metabolic disorder
**562**		1					1	0	0	Metabolic disorder
**563**		1					1	0	0	Metabolic disorder
**564**			1				1	0	0	Lymphoma
**565**		1	1		1		3	0	0	Hepatobiliary functional disorder
**566**		1					1	0	0	Hepatobiliary functional disorder
**567**		1		1			2	0	0	Neurocutaneous disorder
**568**		1					1	0	0	Metabolic disorder
**569**		1					1	0	0	Pancreaticobiliary disorder
**570**		1					1	0	0	Hepatobiliary functional disorder
**571**				2			2	0	0	Tumour
**572**			1	1			2	0	0	Tumour
**573**		1					1	0	0	Metabolic disorder
**574**		1					1	0	0	Metabolic disorder
**575**			2				2	0	0	Tumour
**576**		1					1	0	0	Hepatobiliary functional disorder
**577**				1			1	0	0	Pancreaticobiliary disorder
**578**		1					1	0	0	Hepatobiliary functional disorder
**579**		1					1	0	0	Hepatobiliary functional disorder
**580**			1				1	0	0	Vascular abnormalities and disorder
**581**		1					1	0	0	Hepatobiliary functional disorder
**582**				2			2	0	0	Appendicitis
**583**		1		1			2	0	0	Hepatobiliary functional disorder
**584**		1					1	0	0	Metabolic disorder
**585**		2					2	0	0	Pancreaticobiliary disorder
**586**			2				2	0	0	Neurocutaneous disorder
**587**		1					1	0	0	Pancreaticobiliary disorder
**588**		1					1	0	0	Tumour
**589**			1				1	0	0	Tumour
**590**		1					1	0	0	Pancreaticobiliary disorder
**591**		1					1	0	0	Hepatobiliary functional disorder
**592**			1				1	0	0	Kidney and urological disorder
**593**		1					1	0	0	Tumour
**594**			1				1	0	0	Appendicitis
**595**		1					1	0	0	Pancreaticobiliary disorder
**596**			1				1	0	0	Vascular abnormalities and disorder

**597**		1					1	0	0	Metabolic disorder
**598**		1					1	0	0	Hepatobiliary functional disorder
**599**		1					1	0	0	Metabolic disorder
**600**		1					1	0	0	Hepatobiliary functional disorder
**601**		1					1	0	0	Hepatobiliary functional disorder
**602**			2				2	0	0	Tumour
**603**		1					1	0	0	Neurocutaneous disorder
**604**		6					6	0	0	Vascular abnormalities and disorder
**605**		1					1	0	0	Metabolic disorder
**606**			1				1	0	0	Hepatobiliary functional disorder
**607**		1					1	0	0	Hepatobiliary functional disorder
**608**		1					1	0	0	Hepatobiliary functional disorder
**609**		1					1	0	0	Metabolic disorder
**610**		1					1	0	0	Metabolic disorder
**611**		1					1	0	0	Hepatobiliary functional disorder
**612**		1					1	0	0	Hepatobiliary functional disorder
**613**		1					1	0	0	Hepatobiliary functional disorder
**614**		1					1	0	0	Hepatobiliary functional disorder
**615**		2					2	0	0	Hepatobiliary functional disorder
**616**		1					1	0	0	Pancreaticobiliary disorder
**617**		1					1	0	0	Hepatobiliary functional disorder
**618**		1					1	0	0	Metabolic disorder
**619**		1					1	0	0	Hepatobiliary functional disorder
**620**			2				2	0	0	Tumour
**621**			1	2			3	0	0	Neurocutaneous disorder
**622**		1					1	0	0	Metabolic disorder
**623**		1					1	0	0	Hepatobiliary functional disorder
